# Crossed SMPS MOSFET-based protection circuit for high frequency ultrasound transceivers and transducers

**DOI:** 10.1186/1475-925X-13-76

**Published:** 2014-06-12

**Authors:** Hojong Choi, K Kirk Shung

**Affiliations:** 1 NIH Transducer Resource Center and Department of Biomedical Engineering, University of Southern California, Los Angeles, CA, USA

**Keywords:** Protection circuit, SMPS MOSFET, Ultrasound system, Ultrasonic transducer

## Abstract

**Background:**

The ultrasonic transducer is one of the core components of ultrasound systems, and the transducer’s sensitivity is significantly related the loss of electronic components such as the transmitter, receiver, and protection circuit. In an ultrasonic device, protection circuits are commonly used to isolate the electrical noise between an ultrasound transmitter and transducer and to minimize unwanted discharged pulses in order to protect the ultrasound receiver. However, the performance of the protection circuit and transceiver obviously degrade as the operating frequency or voltage increases. We therefore developed a crossed SMPS (Switching Mode Power Supply) MOSFET-based protection circuit in order to maximize the sensitivity of high frequency transducers in ultrasound systems.

The high frequency pulse signals need to trigger the transducer, and high frequency pulse signals must be received by the transducer. We therefore selected the SMPS MOSFET, which is the main component of the protection circuit, to minimize the loss in high frequency operation. The crossed configuration of the protection circuit can drive balanced bipolar high voltage signals from the pulser and transfer the balanced low voltage echo signals from the transducer.

**Methods:**

The equivalent circuit models of the SMPS MOSFET-based protection circuit are shown in order to select the proper device components. The schematic diagram and operation mechanism of the protection circuit is provided to show how the protection circuit is constructed. The P-Spice circuit simulation was also performed in order to estimate the performance of the crossed MOSFET-based protection circuit.

**Results:**

We compared the performance of our crossed SMPS MOSFET-based protection circuit with a commercial diode-based protection circuit. At 60 MHz, our expander and limiter circuits have lower insertion loss than the commercial diode-based circuits. The pulse-echo test is typical method to evaluate the sensitivity of ultrasonic transducers. Therefore, we performed a pulse-echo test using a single element transducer in order to utilize the crossed SMPS MOSFET-based protection circuit in an ultrasound system.

**Conclusions:**

The SMPS-based protection circuit could be a viable alternative that provides better sensitivity, especially for high frequency ultrasound applications.

## Background

High frequency (>15 MHz) ultrasound systems make it possible to visualize small-size biological structures such as eye, skin, and blood vessel walls due to higher (<mm) spatial resolution at the expense of scarifying penetration depth [1,2]. The performances of these systems are critically affected by solid state electronics, such as the protection circuit and analog front-end transceiver, due to parasitic impedance caused by the interconnection of the electronic components [2]. This is especially true at higher operating frequencies or voltages [3,4]. Additionally, higher frequency transducers typically have lower sensitivity and bandwidth [3]. Therefore, enhancing the performance of the protection circuit and transceiver is of crucial importance for high frequency ultrasound systems and transducers.

The ultrasound analog transceiver typically consists of a pulser as a transmitter and preamplifier as a receiver. Since the pulser drives the high frequency transducer with low sensitivity, high voltage pulse signals with low ring-down are desirable in order to obtain reasonable pulse duration [5]. The preamplifier should have a low noise figure (NF) and wide bandwidth [5]. There are several high-frequency commercial pulsers and receivers. The unipolar pulser/receiver (Panametrics 5900PR, Olympus Inc., Waltham, MA, USA) and bipolar pulser (AVB2-TE-C monocycle generator, Avtech Electro Systems, Ottawa, Ontario, Canada) have been used extensively for ultrasound research [3]. A bipolar pulser is generally preferred due to its accuracy of controlling bandwidth and center frequency, which makes it easier to filter out unwanted noise from the out-of-certain frequency band [5].

Protection circuits consist of passive-type circuits, which have no external biasing capabilities, or active-type circuits, which are required for additional biasing. For passive-type circuits, the commercial diode-based expander (DEX-3, Matec Instruments, Northborough, MA, USA) and diode-based limiter (DL-1, Matec Instruments, Northborough, MA, USA) have been used for ultrasound research [3,5]. For active-type circuits, diode-bridge-based circuits, such as the MD0100DB1 (Supertex, Sunnyvale, CA, USA), are widely used because of their ability to isolate noise between the transmitter and transducer [6]. However, the passive-type protection circuit is often more desirable due to its ability to operate without a DC power supply. The noise signals from DC power supplies could be critical for high frequency transducer array applications [7]. Additionally, the number of connections must be minimized for certain ultrasound array transducer applications, such as intravascular ultrasound imaging.

In order to obtain strong sensitivity in high frequency ultrasound systems, there are desirable characteristics for the protection circuits [5]. The expander circuit allows high voltage pulse signals with low insertion loss (IL), while isolating the noise signals between the pulser and transducer [8]. Meanwhile, the limiter circuit protects the preamplifier from unwanted high voltage discharged pulse signals and allows the low voltage echo signals with less signal loss [8]. We therefore proposed a novel crossed SMPS MOSFET-based protection circuit in order to minimize the signal loss for high frequency ultrasound transducers and transceivers.

For the protection circuit, a SMPS MOSFET was selected in order to minimize the loss at high frequencies, since high frequency pulse signals are required to trigger the transducers, and high frequency echo signals are received by the transducers [3,9,10].

The SMPS MOSFET device originally had different values for the parasitic gate-source and gate-drain capacitances (C_gs_ and C_gd_) as well as the gate and drain resistances (R_g_ and R_d_) [9]. Therefore, we designed the protection circuit to have a crossed structure in order to drive balanced positive and negative amplitude of the bipolar signal.

The loss of the protection circuit should be as low as possible in order to maximize the transducer sensitivity. The gate-drain capacitance is the largest parasitic capacitance of the SMPS MOSFET [11]. To remove this capacitance, gate-drain connected SMPS MOSFET devices are used with series and parallel connections. The series and parallel gate-drain connected configuration can also reduce total internal resistance of the SMPS MOSFET, which improves the IL performance [7].

In the Methods section, we describe the architecture of the crossed SMPS MOSFET-based protection circuit and provide its estimated performance. In the Results and Discussion section, we evaluate and compare the performance of the SMPS MOSFET-based protection circuit with a commercial diode-based protection circuit. We also performed a pulse-echo measurement to validate the capability of the SMPS MOSFET-based protection circuit in an ultrasound system.

## Methods

**Figure 1:**
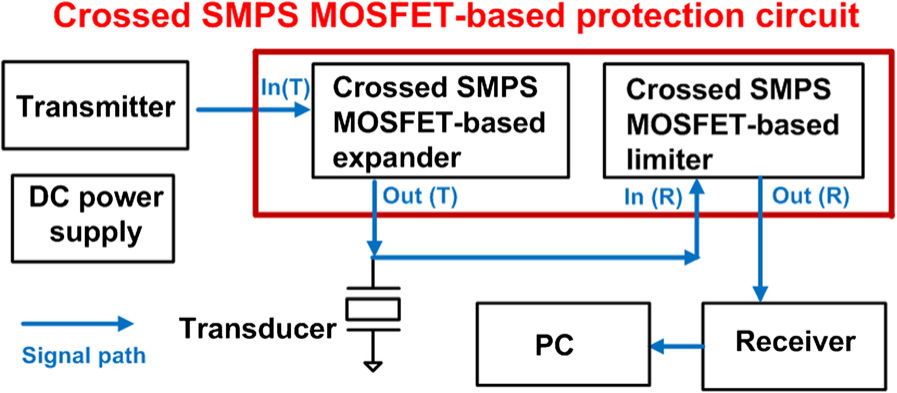
Block diagram of the crossed SMPS MOSFET-based protection circuit with a single element transducer.

Figure 1 illustrates a block diagram of the crossed SMPS MOSFET-based protection circuit with a single element transducer. The output signals of the transmitter trigger the transducer through the crossed SMPS MOSFET-based expander. The returned low voltage echo signals from the transducer and discharged high voltage pulsed signals from the transmitter are transferred to the crossed SMPS MOSFET-based limiter. The limiter circuit clamps the high voltage pulse signals and passes the low voltage echo signals to the receiver. The echo signals amplified by the receiver are transferred to PC.

**Figure 2:**
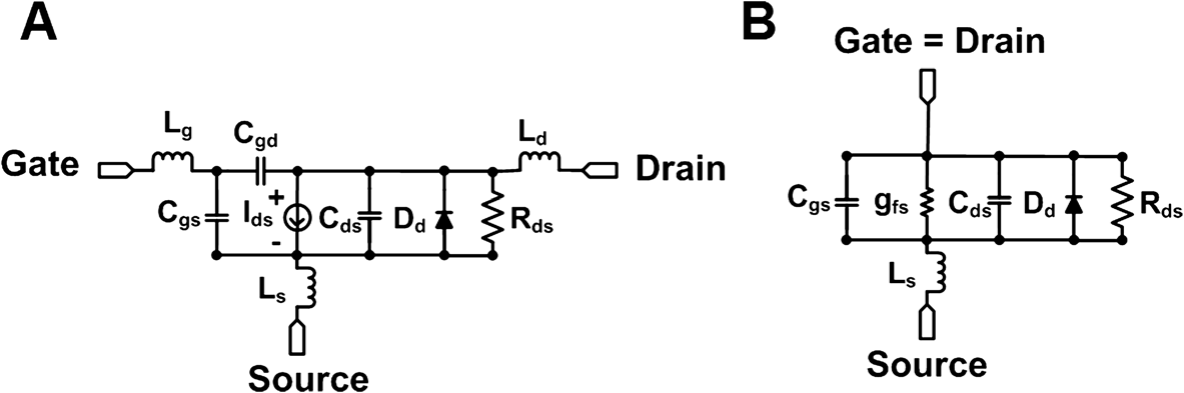
**Equivalent circuit models for the SMPS MOSFET.** The equivalent circuit models of the **(A)** SMPS MOSFET and **(B)** gate-drain connected SMPS MOSFET.

The equivalent circuit model of the SMPS MOSFET can inform us how to select the proper devices to construct the protection circuit. Figure 2A and 2B show the equivalent circuits of the SMPS MOSFET and gate-drain connected SMPS MOSFET [9,11]. Figure 2A illustrates the large signal equivalent circuit model of a SMPS MOSFET device [11,12]. In the equivalent circuit model, there are undesirable parasitic resistance (R_ds_), inductances (L_g_, L_d_ and L_s_), and capacitances (C_gs_, C_gd_ and C_ds_), as well as a drain-source current (I_ds_) and built-in diode (D_d_). Especially, the gate-drain capacitance (C_gd_) is the largest parasitic capacitance and critically deteriorates the bandwidth and recovery time of the devices [11]. Therefore, the gate-drain connected configuration can eliminate that large parasitic capacitance, including the gate and drain inductances (L_g_ and L_d_). As shown in Figure 2B, the MOSFET devices should also have low drain-source resistance (R_ds_) and forward transconductance (g_fs_^-1^) in order to minimize the IL. Therefore, a N-channel SMPS MOSFET device (IRF5801, International Rectifier, El Segundo, CA, USA) was selected as the main element because it can tolerate high voltage signals up to 200V_p-p_ and provide fast turn-on time (<10 ns). The static drain-source resistance and forward transconductance are both very low (2.2 Ω), making this device suitable for low loss, high frequency, and high voltage operation.

**Figure 3:**
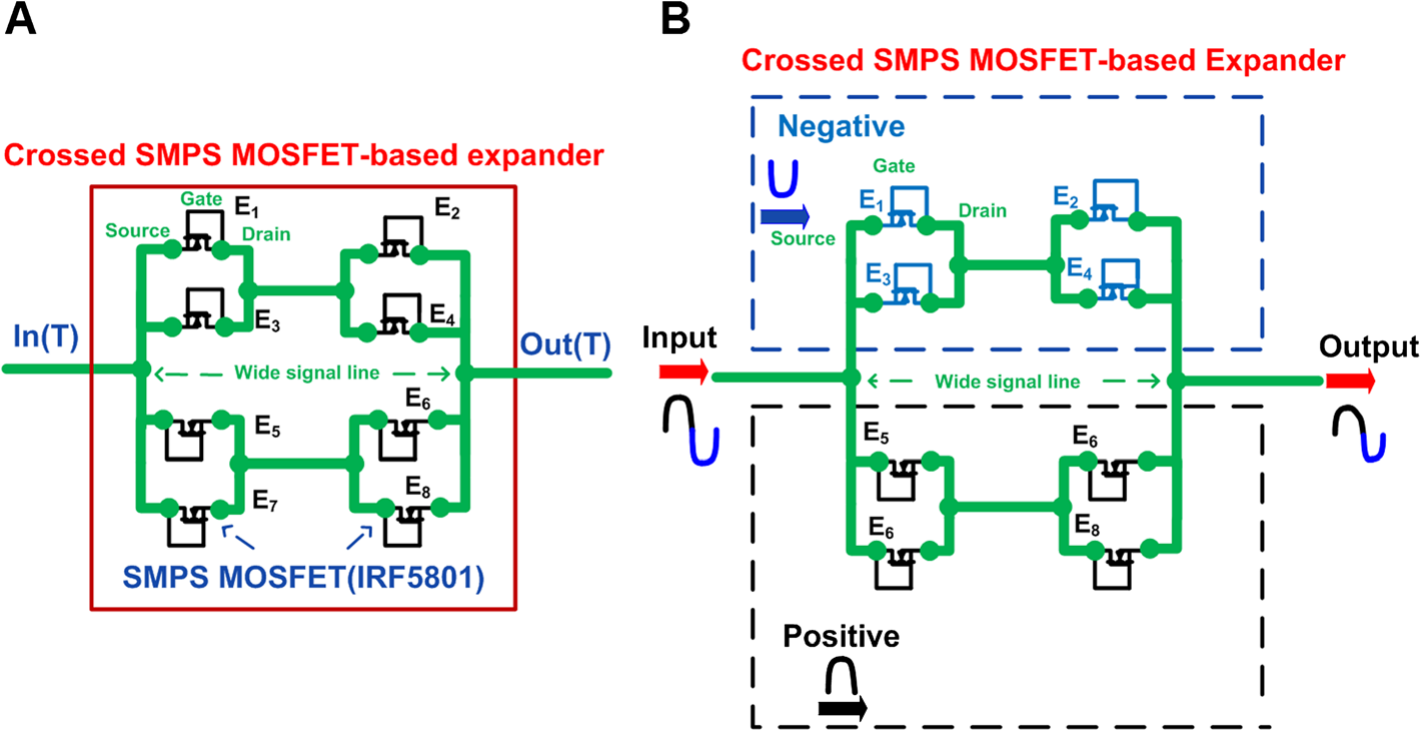
**Architecture of the expander. (A)** Circuit diagram and **(B)** operation mechanism of the crossed SMPS MOSFET-based expander.

Figure 3A illustrates the circuit schematic diagram of the crossed SMPS MOSFET-based expander. It was implemented using eight SMPS MOSFETs (E_1_ ~ E_8_: IRF5801). The MOSFET components were connected by wide signal lines (~0.1 cm width) in order to reduce high voltage stress and distortions to the SMPS MOSFET devices [13]. In the protection circuit, the gate-drain connected MOSFET behaves as a diode-connected MOSFET so that the unipolar (negative or positive) signal passes through the gate-drain MOSFET [14]. As shown in Figure 3B, negative (-) and positive (+) high voltage signals from the pulser pass through the upper (E_1_ ~ E_4_) and lower (E_5_ ~ E_8_) parts of the expander, respectively. The combined high voltage signals then flow to the transducer.

**Figure 4:**
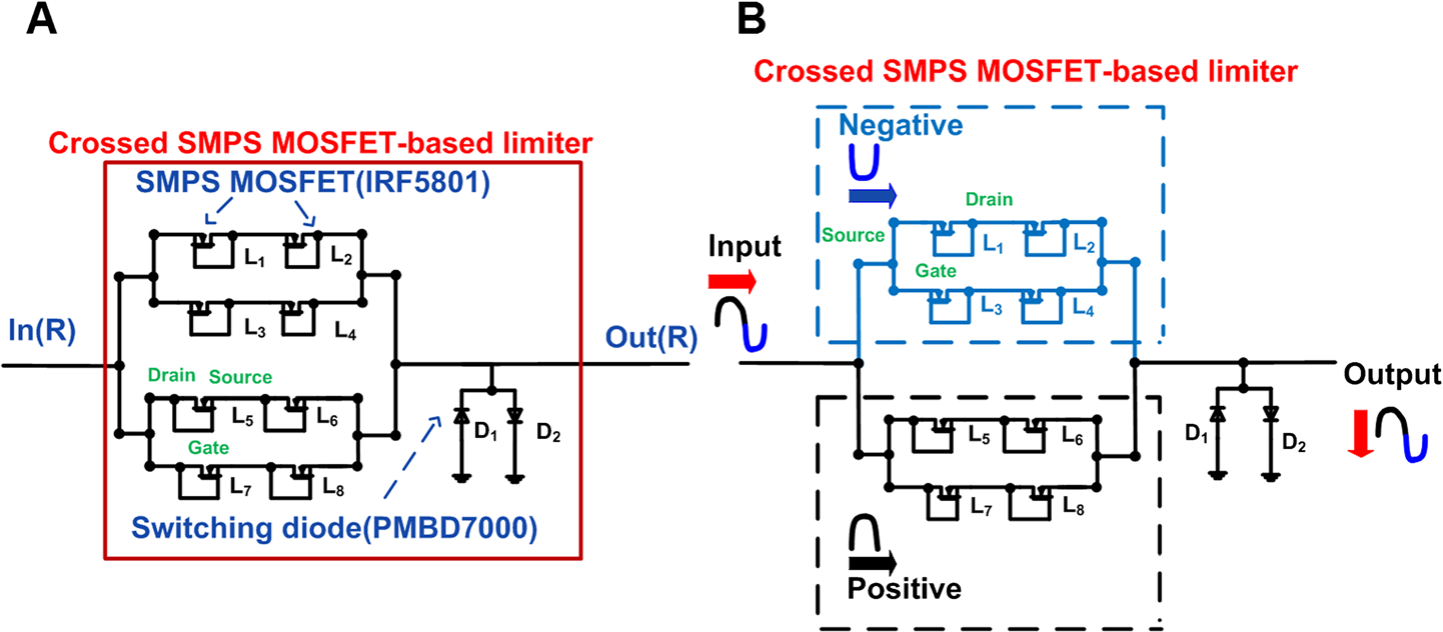
**Architecture of the limiter. (A)** Circuit diagram and **(B)** operation mechanism of the crossed SMPS MOSFET-based limiter.

In Figure 4A, the SMPS MOSFET-based limiter circuit was implemented using an SMPS MOSFET (L_1_ ~ L_8_: IRF5801) and switching diode (D_1_ and D_2_: PMBD7000, NXP Semiconductors, Eindhoven, Netherlands). The switching diode can tolerate high voltages (up to 100 V) and fast reverse recovery time (4 ns), which is appropriate for rapidly clamping the high voltage discharged pulses. Similar to the expander circuit, the SMPS MOSFET-based limiter circuit also utilized a crossed configuration in order to drive the balanced signal. As shown in Figure 4B, the negative and positive high voltage discharged signals pass through the upper part (L_1_ ~ L_4_) and lower part (L_5_ ~ L_8_) of the limiter. Subsequently, combined positive and negative signals pass through the switching diodes (D_1_ and D_2_) to the ground in order to protect the receiver. Meanwhile, the high voltage discharged signals are attenuated slightly by the SMPS MOSFET devices, resulting in decreased high voltage amplitude.

The P-Spice circuit program (Cadence Design System, San Jose, CA, USA) was used to estimate the performance of the crossed SMPS MOSFET-based expander and limiter. The IL of the devices can be calculated as

(1) $${\mathrm{IL}}_{\text{expander}}=20\cdot \mathrm{Log}\;\left(\frac{\text{output}\;\text{peak}\;\text{to}\;\text{peak}\;\text{amplitude}\;\text{with}\;\text{the}\;\text{device}}{\text{output}\;\text{peak}\;\text{to}\;\text{peak}\;\text{amplitude}\;\text{without}\;\text{the}\;\text{device}}\right)$$

The RT of the limiters is

(2) $$\mathrm{RT}=T_{f}-T_{s}\;$$

where T_f_ is the time for the discharged output pulse of the limiter to reach at the ±1% point of the final discharged output pulse of the limiter, and T_s_ is the time for the first discharged output pulse of the limiter to start to rise/fall at the ±1% point of the first output pulse.

The expected ILs of the crossed SMPS MOSFET-based expander and limiter are -0.95 and -1.3 dB at 60 MHz, respectively. The peak-to-peak voltage and RT of the SMPS MOSFET-based limiter using a 60 MHz 70V_p-p_ signal are 3.3 V_p-p_ and 120 ns, respectively. However, the simulation data for the SMPS MOSFET devices operating at high frequency and voltage are not accurate for predicting the behavior of the protection circuits [15,16]. It is reported here merely for the purpose of estimating the performance of the crossed SMPS MOSFET-based expander and limiter. Additionally, the library of commercial protection circuits is not available from the manufacturer, so the expected data are not provided in this paper.

## Results and discussion

### Crossed SMPS MOSFET-based expander

**Figure 5:**
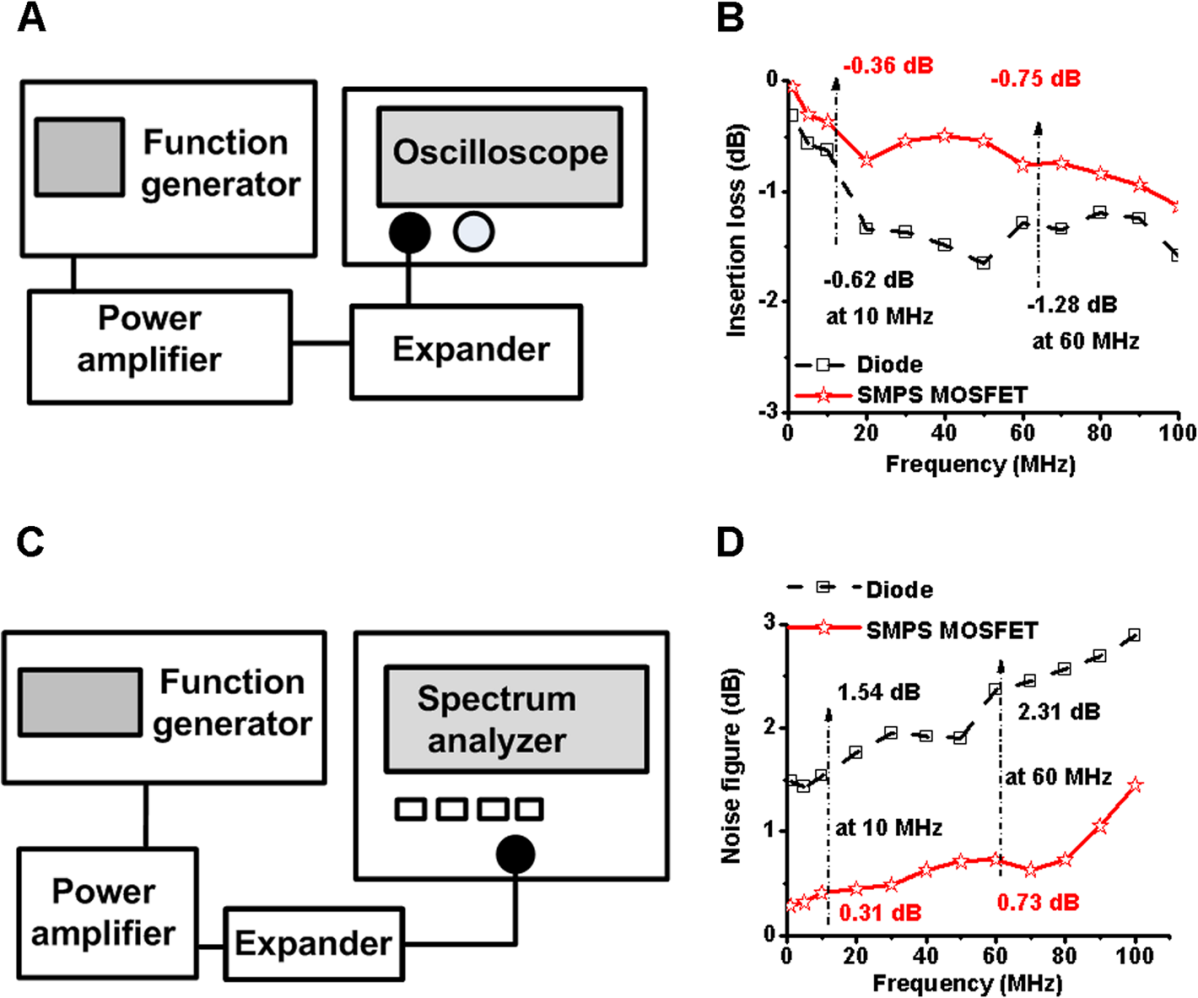
**Experimental measurements for the expanders. (A)** The experimental arrangement used to measure the IL of the expanders, **(B)** IL of the expanders when a 70 V_p-p_ pulsed sine waveform from the power amplifier was applied, **(C)** experimental arrangement for the NF measurement and **(D)** NF of the expanders. “Diode” refers to the commercial diode-based expander (DEX-3) and “SMPS MOSFET” refers to the crossed SMPS MOSFET-based expander.

The IL of the SMPS MOSFET-based and commercial diode-based expanders (DEX-3) was measured in order to evaluate and compare their performance. Figure 5A shows the experimental arrangement of the expander circuits used to measure the IL. The single pulse signal from the function generator (AFG2020, Tektronix, Beaverton, OR, USA) was sent to the power amplifier (325LA, E&I, Rochester, NY, USA). A 70V_p-p_ pulsed signal was sent to the expanders, and the output signal of the expanders was displayed on an oscilloscope (LC-534, LeCroy, Chesnutt Ridge, NY, USA). As shown in Figure 5B, the IL of the crossed SMPS MOSFET-based expander (-0.36 dB and -0.75 dB at 10 MHz and 60 MHz, respectively) is lower than that of the commercial diode-based expander (-0.62 dB and -1.28 dB at 10 MHz and 60 MHz, respectively). This is true over the entire 1 MHz to 100 MHz range.

The NF of an electronic component indicates how much the component affects the overall system noise level [17]. We measured the NF of the expanders in order to estimate their noise isolation capabilities. Figure 5C shows the experimental arrangement used to measure the NF of the expanders. The “Gain Method” was used in order to obtain the NF data of the expanders with a function generator, power amplifier and spectrum analyzer (E4401B, Agilent Technologies, Santa Clara, CA, USA) [18]. The NF of the expander (NF_expander_) can be calculated as

(3) $${\mathrm{NF}}_{\text{expander}}=\frac{{\mathrm{NF}}_{\text{poweramp}+\text{expander}}-{\mathrm{NF}}_{\text{poweramp}}}{{\mathrm{IL}}_{\text{expander}}}+1$$

where NF_poweramp+expander_ is the NF of the power amplifier and expander, NF_poweramp_ is the NF of the power amplifier, and IL_expander_ is the IL of the expander.As shown in Figure 5D, the NF of the SMPS MOSFET-based expander (0.31 dB and 0.73 dB at 10 MHz and 60 MHz, respectively) is lower than that of the diode-based expander (1.54 dB and 2.31 dB at 10 and 60 MHz, respectively). The measurement data confirms that the SMPS MOSFET-based expander is better at isolating noise than the diode-based expander.

### Crossed SMPS MOSFET-based limiter

**Figure 6:**
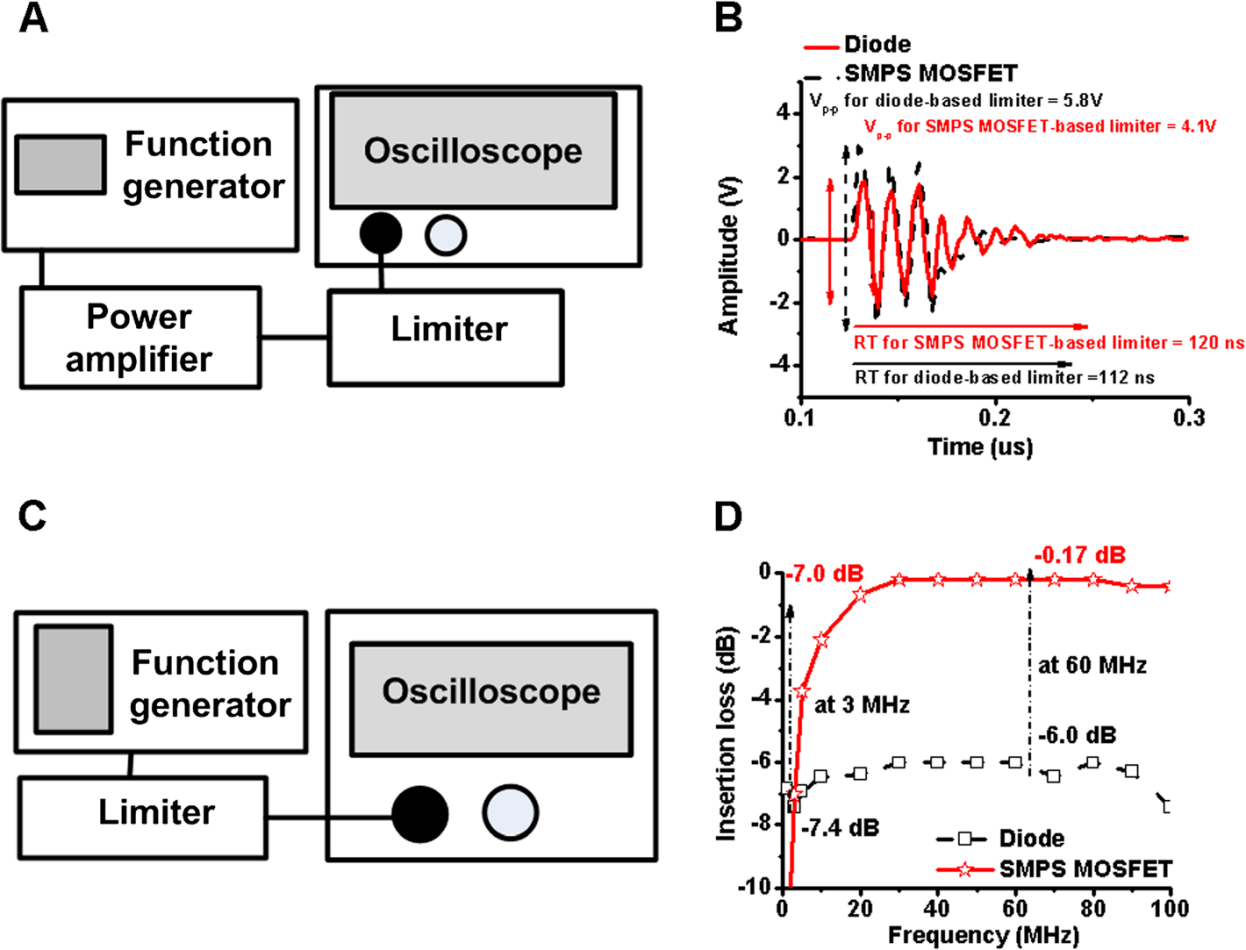
**Experimental measurements for the limiters. (A)** The experimental arrangement used to measure the high voltage blocking capability of the limiters, **(B)** suppressed output waveforms and RT of the limiters when a three-cycle 60 MHz, 70 V_p-p_ pulsed sine waveform from the power amplifier was applied, **(C)** experimental arrangement for the IL measurement, and **(D)** IL of the limiters when a 200 mV_p-p_ pulsed sine waveform from the function generator was applied. “Diode” refers to the commercial diode-based limiter (DL-1) and “SMPS MOSFET” refers to the crossed SMPS MOSFET-based limiter.

Figure 6A shows the experimental setup used to measure the high voltage blocking capability of the crossed SMPS MOSFET-based limiter. A 60 MHz, three cycle sine-wave signal was delivered from the function generator (AFG3251, Tektronix, Beaverton, OR, USA) to the power amplifier (325LA). The 60 MHz 70V_p-p_ output signal from the power amplifier was delivered to the limiter to test the high voltage blocking capability, since the discharged pulse signals could be several cycles (even though the pulser typically sends only a single-cycle pulse to the transducer for covering the transducer bandwidth). The output waveform of the limiters was recorded on a oscilloscope (LC534) and processed on a PC using LabVIEW (National Instrument, Austin, TX, USA). As illustrated in Figure 6B, the output signal amplitude of the crossed SMPS MOSFET-based limiter (4.1 V) is lower than that of the commercial diode-based limiter (5.8 V).

The RT of the crossed SMPS MOSFET-based limiter (120 ns) is slightly longer than that of the diode-based limiter (112 ns). However, the discharged voltages and RTs of both limiters are acceptable for high frequency transducer applications.

In order to estimate the signal loss in the low voltage operation, IL performances of the limiters were obtained from 1 MHz to 100 MHz. Figure 6C shows the experimental setup to measure the IL of the limiters. The function generator (AFG3251) delivered 60 MHz, 200 mV_p-p_ pulsed sine wave signals to the limiters and the output waveforms of the limiters were displayed on an oscilloscope (LC534) in order to calculate the IL. As shown in Figure 6D, the IL of the crossed SMPS MOSFET-based limiter (-7.0 dB) is slightly lower than that of the diode-based limiter (-7.4 dB) at 3 MHz. However, the IL of the crossed SMPS MOSFET-based limiter (-0.17 dB) is substantially lower than that of the diode-based limiter (-6.0 dB) at 60 MHz. Additionally, the crossed SMPS MOSFET-based limiter provides flatter loss than the commercial diode-based limiter in the wide frequency range (>20 MHz). Therefore, the crossed SMPS MOSFET-based limiter is capable of further reducing the IL at high frequency operation.

### Pulse-echo measurement

**Figure 7:**
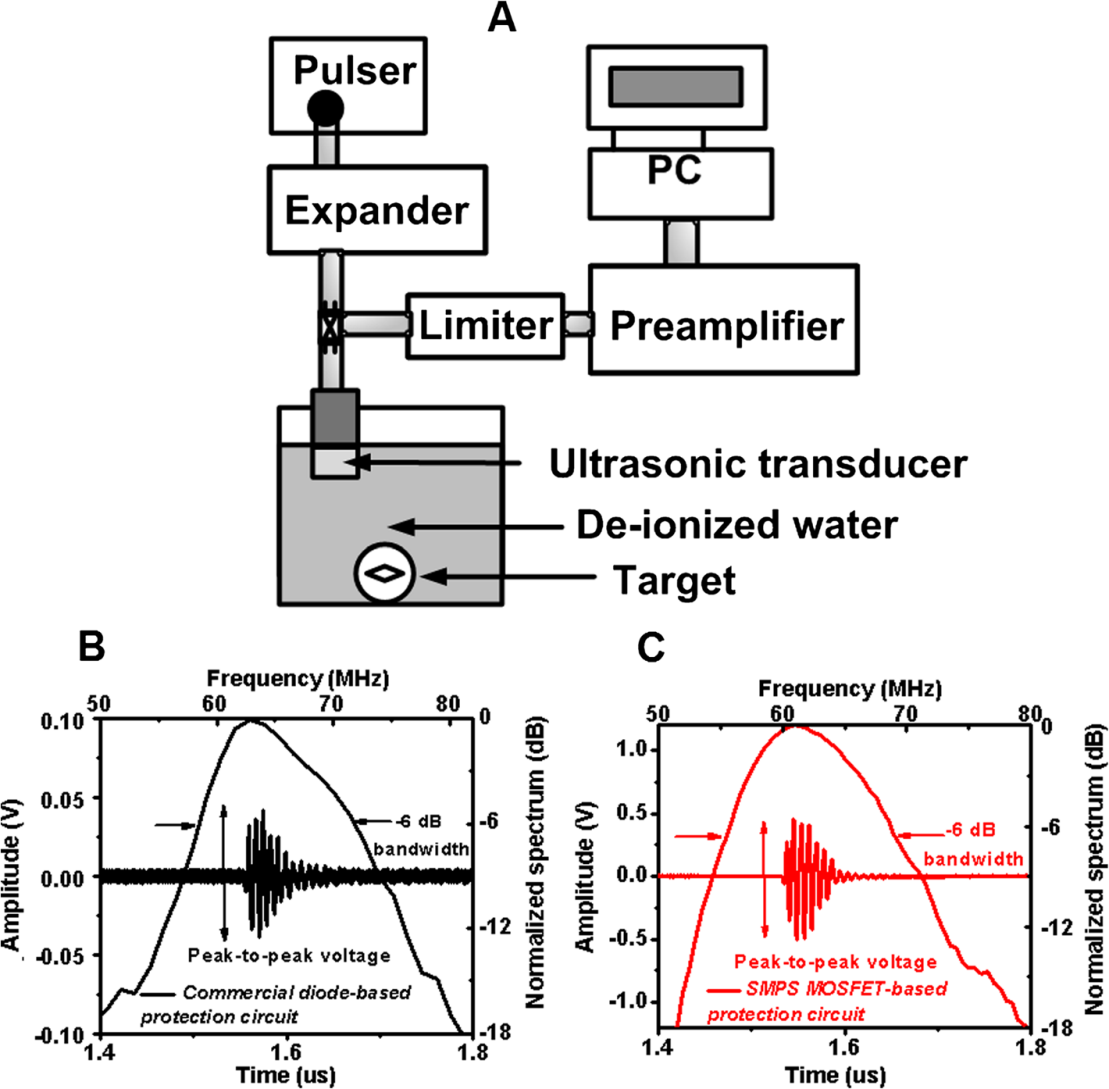
**Pulse-echo measurements with the protection circuits. (A)** The experimental setup used for the pulse-echo test. Pulse-echo response comparisons between **(B)** the commercial protection circuit and **(C)** crossed SMPS MOSFET-based protection circuit with a pulser and receiver.

The crossed SMPS MOSFET-based protection circuit was designed to maximize sensitivity in high frequency ultrasonic applications. Therefore, the typical pulse-echo measurement was performed with the transceiver and transducer in order to determine the electronic device’s performance in high frequency ultrasound systems. Figure 7A illustrates the experimental diagram used for the pulse-echo measurement for evaluating and comparing the performance of our protection circuit with commercial protection circuits (DL-1 and DEX-3). The commercial pulser (AVB2-TE-C) was selected due to its capability to generate a bipolar pulse with reasonable flat bandwidth in the desired high frequency ranges. Figure 7B and 7C compare the echo signal responses of the crossed SMPS MOSFET-based circuit to those of the commercial diode-based protection circuits, using a commercial pulser (AVB2-TE-C) and preamplifier (OPA846). The preamplifier was the typical non-inverting operational amplifier (OPA846, Texas Instrument, Dallas, TX, USA) with resistive feedback, which can provide 18-dB voltage gain and 120 MHz -3 dB bandwidth. The OPA846 was selected because of its low input-referred noise (1.2 nV/√Hz). The single element 60 MHz LiNbO_3_ (Lithium Niobate) ultrasonic transducer was fabricated in our laboratory. Pulse-echo tests were performed with the ultrasonic transducer with a 2.2 mm aperture size and 2.2 mm focal distance. The transducer was focused to a quartz target in deionized water. A 60 MHz, a 70 V_p-p_ single bipolar pulse generated by the pulser was sent to trigger the transducer through the expander, and the returned echo signals were amplified by the preamplifier and transferred to the PC. The RF data was processed using Fast Fourier Transform to obtain the spectral data.

The peak-to-peak amplitude of the echo signals received by the transducer using our protection circuit is 9.4 times larger than that of the commercial protection circuit due to the lower IL of the expander and limiter (-0.75 and -0.17 dB at 60 MHz). The -6 dB bandwidth of echo spectrum generated by the transducer using our circuit is slightly wider (5.7%) than that of the commercial diode-based protection circuit. These pulse-echo measurement results confirm that the crossed SMPS MOSFET-based protection circuit provides better sensitivity than the commercial diode-based protection circuit. The pulse-echo measurement data show that the highest sensitivity was achieved among the currently developed protection circuits because the SMPS MOSFET-based protection was designed to minimize the signal loss and provide broad flat bandwidth. Therefore, the proposed architecture could be useful in applications involving high frequency ultrasonic transducers that have very low sensitivity.

## Conclusion

We report a novel crossed SMPS MOSFET-based protection circuit for high frequency transceivers and transducers. We compared its performance to commercial diode-based protection circuits. The crossed SMPS MOSFET-based expander, which uses a wide signal line, produces better IL and NF (-0.75 and 0.73 dB) than the commercial diode-based expander (-1.28 and 2.31 dB) at 60 MHz. The crossed SMPS MOSFET-based limiter also produces lower IL and discharge voltage (-0.17 dB and 4.1 V) than the commercial diode-based limiter (-6.0 dB and 5.8 V) at 60 MHz. We tested the protection circuit with a 60 MHz ultrasonic transducer in order to confirm device performance. The proposed protection circuit shows an echo signal 9.4 times larger than that of the commercial diode-based circuit and is therefore useful for high frequency ultrasound applications with very low sensitivity.

## Abbreviations

SMPS: Switching mode power supply; IL: Insertion loss; NF: Noise figure; LiNbO_3_: Lithium niobate; RT: Recovery time.

## Competing interests

The authors declare that they have no competing interests.

## Authors’ contributions

HC proposed the idea, performed the experiments and wrote the manuscript. KKS suggested guidance of the project, discussed and reviewed the results, and revised the manuscript. Both authors read and approved the final manuscript.
